# In vitro and in vivo activity of GT-1, a novel siderophore cephalosporin, and GT-055, a broad-spectrum β-lactamase inhibitor, against biothreat and ESKAPE pathogens

**DOI:** 10.1038/s41429-021-00472-9

**Published:** 2021-09-14

**Authors:** Stephanie A. Halasohoris, Jennifer M. Scarff, Lisa M. Pysz, Sanae Lembirik, Margaret M. Lemmon, Donald Biek, Brendan Hannah, Steven D. Zumbrun, Rekha G. Panchal

**Affiliations:** 1 US Army Medical Research Institute of Infectious Diseases, Fort Detrick, MD USA; 2 Geom Therapeutics Inc, San Diego, CA USA

**Keywords:** Antibiotics, Drug screening

## Abstract

Antimicrobial-resistance (AMR) has become an increasingly difficult issue to overcome for bacteria associated with both community- and hospital-acquired infections as well as potential biodefense threats. The need to identify new therapeutics of novel classes and/or with unique mechanisms is critical to combatting AMR in the coming years. GT-1 (LCB10-0200), a siderophore-linked cephalosporin, is one such novel option and is formulated to be used either alone or in combination with a novel broad-spectrum β-lactamase inhibitor, GT-055 (LCB18-055). This study assessed the in vitro and in vivo efficacy of GT-1 and GT-055 against a broad array of multi-drug resistant and biothreat pathogens. Here, we demonstrated sub-4 µg ml^−1^ efficacy against a number of pathogens in vitro. We further determined that in mice infected via aerosol route with *Yersinia pestis*, efficacy of GT-1/GT-055 treatment is at least equivalent to the comparator antibiotic, ciprofloxacin.

## Introduction

The CDC estimates that there are over 2.8 million infections that result in almost 36,000 deaths from drug-resistant bacteria and fungi per year [1]. It has been over a decade since attention was brought to the need for new therapeutics against the difficult-to-treat ESKAPE (*Enterococcus faecium*, *Staphylococcus aureus*, *Klebsiella pneumoniae*, *Acinetobacter baumannii*, *Pseudomonas aeruginosa*, and *Enterobacter* species) pathogens [2, 3]. And yet, in a 2019 CDC report, multiple drug-resistant (MDR) infections increased in frequency from 2012 to 2017, including extended spectrum β-lactamase (ESBL) producing Enterobacterales (e.g., *K. pneumoniae* and *E. coli*). The CDC ranks drug-resistant bacteria as urgent, serious, or concerning threats and four β-lactam antibiotic-resistant pathogens are on these lists. Carbapenem-resistant Enterobacterales and carbapenem-resistant *Acinetobacter* were ranked as urgent threats while multidrug-resistant *P. aeruginosa* and ESBL-producing Enterobacterales were ranked as serious threats [1]. This highlights the need for new antibiotics or combinations that circumvent these resistance mechanisms.

In addition to the ESKAPE pathogens, infections by biodefense pathogens would also benefit from improved treatments. *Burkholderia pseudomallei* infections are particularly difficult to treat, with a months-long treatment regimen for which failures are not uncommon. *B. pseudomallei* has a reduced membrane permeability compared to other Gram-negative bacteria. The resistance to β-lactam antibiotics can be mediated through the PenA β-lactamase or through mutations and gene rearrangements [4]. These acquired resistances have a low frequency of occurrence, but given the low number of available antibiotics to treat *B. pseudomallei infections*, they remain an issue of concern. Infections by another biodefense pathogen, *Yersinia pestis* have a small window for initiation of effective treatment and there exist only a few recommended antibiotics [5].

GT-1 (LCB10-0200) is a novel therapeutic that consists of a siderophore, dihydroxypyridone, conjugated to a modified aminothiazoylglycyl cephalosporin [6]. The goal of this approach is to exploit the bacterial siderophore uptake systems to enable antibiotic entry in a “Trojan horse” strategy, similar to the strategy successfully employed by cefiderocol [7]. In addition, the conjugated molecule is resistant to hydrolysis by many ESBLs and carbapenemases. GT-1 was more effective against clinical isolates of *P. aeruginosa*, *Klebsiella oxytoca*, *Proteus* spp., *Serratia marcescens*, and *Enterobacter aerogenes* than ceftazidime and ceftriaxone. This improved activity was also observed against β-lactamase-producing strains of *P. aeruginosa* [6]. GT-1 also resulted in a larger reduction in bacterial load than ceftazidime when used to treat four strains of *P. aeruginosa* in murine systemic and thigh infection models [6].

**Fig. 1:**
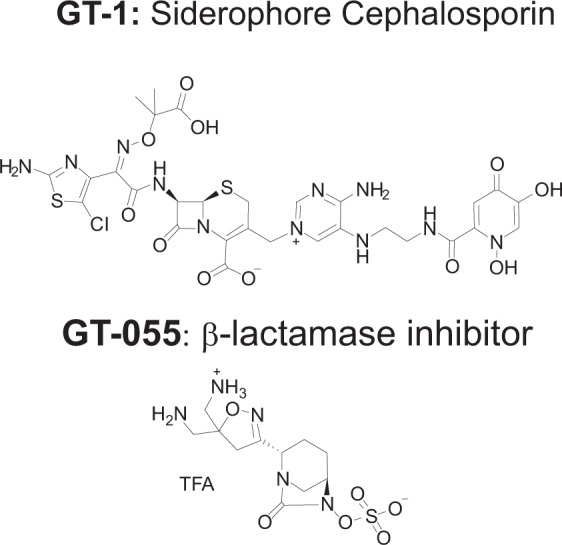
Chemical structures of GT-1 (LCB10-0200) and GT-055 (LCB18-055)

β-lactam antibiotics are often coupled with a β-lactamase inhibitor as a way to counteract the enzymes produced by resistant bacteria. To that effect, the efficacy of GT-1 when coupled with GT-055 (LCB18-055), a novel β-lactam inhibitor (BLI), was also investigated [8]. The chemical structures of both GT-1 and GT-055 are shown in Fig. 1. The GT-1/GT-055 combination was tested against *E. coli*, *K. pneumoniae*, and *Acinetobacter* spp. isolates with a variety of β-lactamase profiles. Against all three bacterial genera, GT-1 alone had lower MICs against most strains than ceftazidime alone. GT-1/GT-055 in combination had reduced MICs against many strains compared to GT-1 alone and was better than the combination of ceftazidime/avibactam, particularly against *E. coli* and *K. pneumoniae*. It has been found that cefiderocol is active in the presence of many β-lactam hydrolyzing enzymes, even without the addition of a β-lactamase inhibitor [9, 10].

Pharmacokinetic (PK) studies of GT-1 in mouse, rat and dog have been reported. The PK parameters of GT-1 across different species suggest low clearance, low volume of distribution, and a short half-life, with a high free drug fraction [11]. Dose-fractionation studies demonstrated that the PK/Pharmacodynamic (PD) index associated with GT-1 efficacy in the mouse thigh model is plasma free-drug %T > MIC. The free-drug %T > MIC associated with net bacterial stasis, 1-log kill, and 2-log kill are 40.2, 58.3, and 85.8, respectively for *Enterobacterales* and *P. aeruginosa* [12]. Studies of GT-1 efficacy in a mouse lung infection model have also been performed and PK/PD targets determined [13]. The PK characteristics of GT-055 are similar to those of GT-1, and dose-fractionation studies demonstrated that the PK/PD index associated with GT-055 efficacy in a one-compartment in vitro infection model is plasma free-drug AUC/MIC ratio. For *Enterobacterales*, the free-drug AUC/MIC ratios associated with net bacterial stasis, 1-log kill, and 2-log kill are 4.13, 13.7, 57.2, respectively [14]. Initial PK/PD studies found the optimal GT-1:GT-055 ratio to be 1:1.

Here, we further characterize the activity of GT-1 and GT-055 against MDR bacteria, particularly those with carbapenem and other β-lactam resistances. We investigate the in vitro efficacy of these compounds against the biodefense pathogens *Bacillus anthracis*, *Burkholderia mallei*, *B. pseudomallei*, *Francisella tularensis*, and *Y. pestis*. GT-1 and GT-1/GT-055 were tested in mouse infection models of *B. pseudomallei* and *Y. pestis*. The combination had similar efficacy as the therapeutic standard, ciprofloxacin, in the *Y. pestis* model, although it was less active in the *B. pseudomallei* infection model.

## Materials and methods

### Compounds and bacterial strains

GT-1 and GT- 055 were provided by Legochem Biosciences (Seoul, South Korea). The activity and structures have been previously published for both GT-1 [6] and GT-055 [8]. The AR Bank resistant panels used here, Enterobacterales Carbapenem Breakpoint, Gram-negative Carbapenemase Detection, EnterobacteralesCarbapenemase Diversity, and Isolates with New or Novel Antibiotic Resistance panels were obtained from the CDC (Atlanta, GA). The *B. anthracis*, *B. mallei*, *B. pseudomallei*, *F. tularensis*, and *Y. pestis* strains were obtained from the Unified Culture Collection at USAMRIID (Frederick, MD). The control strains for MIC determination, *S. aureus* (ATCC 29213), *E. coli* (ATCC 25922), and *P. aeruginosa* (ATCC 27853) were obtained from the ATCC (Manassas, VA).

### MIC determination for bacteria from CDC resistance panels

The minimum inhibitory concentration (MIC) of GT-1 and GT-055 either alone or in combination was determined for bacteria in the following panels obtained from the Centers for Disease Control and Prevention (CDC, USA) and U.S. Food and Drug Administration (FDA) Antibiotic Resistance Isolate Bank: The entirety of the Enterobacterales Carbapenem Breakpoint, Gram-negative Carbapenemase Detection, and Enterobacterales Carbapenemase Diversity panels, and select isolates from the collection of New or Novel Antibiotic Resistance panel. The β-lactamase resistance genes in the strains of these panels was obtained from the CDC AR Bank [15]. The control strains for the assays were obtained from the ATCC: *Escherichia coli* (ATCC 25922), *Staphylococcus aureus* (ATCC 29213), and *Pseudomonas aeruginosa* (ATCC 27853).

The MICs were determined in accordance with CLSI guidelines [16, 17]. The CDC resistance panel strains were grown on Mueller Hinton agar overnight at 37 °C. Bacterial colonies were transferred into PBS with a cotton swab and the OD_600_ of the suspension was determined. The amount of bacteria to add to the MIC inoculum was calculated based on the approximated CFU ml^−1^ of an OD_600_ = 1 for each strain and the final concentration of bacteria in the assay, 5 × 10^5^ CFU ml^−1^. The bacteria were diluted into cation-adjusted Mueller Hinton broth (CAMHB) and added to the 96-well plates that contained the serial dilutions of the compounds. Plates were incubated at 37 °C with 5% CO_2_ for 16–20 h. The MIC was determined as the lowest concentration of compound that inhibited growth of the bacteria.

### MIC determination in iron-depleted media

The MICs for the compounds against bacteria grown in iron-depleted CAMHB (ID-CAMHB) were compared to the MICs determined in CAMHB for certain strains. To deplete the iron, 150 g Chelex (Bio-Rad Laboratories) was added per 150 ml CAMHB. After 2 h at room temperature, the medium was filtered with a 0.2 μm filter. The cation levels were adjusted such that the final concentrations were: Ca^2+^(20–25 mg l^−1^); Mg^2+^(10–12.5 mg l^−1^); Zn^2+^(10 μM, ZnSO_4_) and the pH was adjusted to 7.2–7.4, if necessary. The medium was filtered with a 0.2 μm filter again after these adjustments to ensure sterility.

*B. mallei* and *B. pseudomallei* were grown on chocolate agar at 35 °C for 24 h, *F. tularensis* was grown on chocolate agar at 35 °C for 48 h, and *Y. pestis* was grown on sheep blood agar at 28 °C for 24 h. Other strains tested, including the QC strains (*E. coli* ATCC 25922, *S. aureus* ATCC 29213, *P. aeruginosa* ATCC 27853), were grown on sheep blood agar at 35 °C for 24 h. Bacteria were resuspended to 0.5 McFarland and diluted 1:100 in media (CAMHB or ID-CAMHB). The medium for *F. tularensis* was supplemented with 2% IsoVitalex (BD). A volume of 50 μl of the diluted bacteria were added to the microtiter plates for a final concentration of 5 × 10^5^ CFU ml^−1^ in 100 μl. The plates were incubated at 35 °C for 18–24 h (48 h for *F. tularensis*) and the MICs were recorded. Representative strains were used for initial susceptibility experiments and then 30 strains of *B. pseudomallei*, *B. mallei*, and *Y. pestis* were used for determination of MIC_90_s.

### In vivo efficacy

Animal research at the United States Army Medical Research Institute of Infectious Diseases (USAMRIID) was conducted under an animal use protocol approved by the USAMRIID Institutional Animal Care and Use Committee in compliance with the Animal Welfare Act, PHS Policy, and other Federal statutes and regulations relating to animals and experiments involving animals. The facility is accredited by the Association for Assessment and Accreditation of Laboratory Animal Care International and adheres to principles stated in the Guide for the Care and Use of Laboratory Animals (National Research Council, 2011).

Six- to eight-week old BALB/c mice were challenged with *B. pseudomallei* strain 1026b via whole body aerosol. It is possible that re-infection could potentially occur by mice licking their fur, although we have no evidence for this. The aerosol was generated with a three-jet collison nebulizer [18]. All aerosol procedures were controlled and monitored by the Automated Bioaerosol Exposure system [19] operating with a whole-body rodent exposure chamber. Integrated air samples were collected from the chamber during each exposure into an all-glass impinger (AGI). A sample from the AGI was serially diluted and plated on SBA to determine the concentration of bacteria. The challenge dose (CFU per mouse) of *B. pseudomallei* which corresponds to the dose introduced in the chamber was estimated using Guyton’s formula [20]. Since not all mice could be aerosol challenged in a single spray, mice from each run were randomized into the treatment groups (*n* = 10 mice) such that each group contained a similar number of mice from each spray. The mean deposited dose for *B. pseudomallei* was 4.09 × LD_50_s (3 separate sprays of 5.56, 3.49, and 3.22 × LD_50_s). Treatment was initiated at 12 h post-infection and 0.2 ml was administered via either intraperitoneal (IP) or subcutaneous (SC) injection. The treatment groups were: saline (IP); ceftazidime (300 mg kg^−1^; IP); one of three doses of GT-1 (30, 60, or 120 mg kg^−1^; SC); or one of three doses of GT-1/GT-055 at 1:1 ratio (30/30, 60/60, or 120/120 mg kg^−1^; SC). Two additional groups of mice had treatment initiated at 6 h post-infection: GT-1 (120 mg kg^−1^, SC) and GT-1/GT-055 1:1 ratio (120/120 mg kg^−1^; SC). Treatment was q6h and continued for 21 days. Mice were closely monitored for morbidity and moribund mice were euthanized. At day 55 post-infection, the surviving mice were euthanized and necropsies completed to harvest the spleens. The spleens were homogenized and serially diluted for enumeration of the bacterial load.

Six- to eight-week old BALB/c were challenged with *Y. pestis* strain CO92 by intranasal inoculation. The mice were anesthetized with 0.1 ml ketamine–acetylpromazine–xylazine (6.7 mg ml^−1^, 0.1 mg ml^−1^, 0.7 mg ml^−1^, respectively) and 20 μl of bacteria were instilled into a single nare. The challenge dose was 2.9 × 10^4^ CFU (110X LD_50_) [21]. Treatment was initiated at 6 h post infection and consisted of the groups administered in 0.2 ml: saline (SC; q6h); Ciprofloxacin (30 mg kg^−1^; IP; q12h); one of three doses of GT-1 (20, 60, or 200 mg kg^−1^; SC; q6h); or one of three doses of GT-1/GT-055 at 2:3 ratio (20/30, 60/90, or 200/300 mg kg^−1^; SC; q6h) with 10 mice per group. Treatment continued for 7 days post-infection and mice were monitored for signs of morbidity for at least 30 days. Moribund mice were euthanized. After day 30, up to 3 surviving mice per group were euthanized and necropsies completed to remove the lungs and spleens. The organs were homogenized in PBS and serially diluted for enumeration of bacterial load.

## Results

### MIC of GT-1 and GT-055 against AMR bacteria

GT-1 and GT-055 were tested alone and in combination (at 1:1 and 2:1 ratios, respectively) against four panels from the CDC and FDA Antibiotic Resistance Isolate Bank (Supplementary Table 1). Three of the panels were the Enterobacterales Carbapenem Breakpoint, Gram-negative Carbapenemase Detection, and Enterobacterales Carbapenemase Diversity sets. Select strains from the Novel Antibiotic Resistance panel were also used for antimicrobial susceptibility testing. The majority of the strains were not sensitive to GT-055; only 28/168 had an MIC that was ≤ 4 μg ml^−1^. Likewise, only 52/168 strains had MICs ≤ 4 μg ml^−1^ for GT-1 alone. The combinations of GT-1 and GT-055 had improved MICs compared to each compound alone. For the 1:1 ratio, 114/168 strains had MICs ≤ 4 μg ml^−1^ (Table 1) and for the 2:1 ratio, 99/168 strains had MICs ≤ 4 μg ml^−1^ (value is for concentration of GT-1). GT-055 has intrinsic antibacterial activity against some isolates, and the increase in number of isolates inhibited at ≤ 4 µg ml^−1^ for the 1:1 ratio compared to 2:1 may reflect the combination of increased intrinsic activity at higher GT-055 concentrations and inhibition owing to inactivation of β-lactamases in the presence of GT-1.

**Table 1:** Summary of MIC to GT-1/GT-055

	Number of strains with MIC			
Bacteria	≤4/4 μg/ml	8/8–32/32 μg/ml	>32/32 μg/ml	Total
*Acinetobacter baumannii*	4	4	6	14
*Citrobacter species*	5	1	1	7
*Enterobacter aerogenes*	4	2	0	6
*Enterobacter cloacae*	14	2	0	16
*Escherichia coli*	28	3	3	34
*Klebsiella* spp. (not *pneumoniae*)	5	0	0	5
*Klebsiella pneumoniae*	34	3	14	51
*Kluyvera ascorbate*	1	0	0	1
*Morganella morganii*	0	1	1	2
*Proteus mirabilis*	2	1	2	5
*Providencia* spp.	1	1	0	2
*Pseudomonas aeruginosa*	6	3	3	12
*Raoultella ornithinolytica*	1	0	0	1
*Salmonella* spp.	1	1	0	2
*Serratia marcescens*	7	0	2	9
*Shigella sonnei*	1	0	0	1
**Total**	**114**	**22**	**32**	**168**

We analyzed the strains that were not sensitive to 1:1 GT-1:GT-055 (MIC ≥ 8 μg ml^−1^) to determine if particular bacterial genera or the presence of resistance genes resulted in an increased likelihood of resistance. *A. baumannii* had the highest percentage of strains that were not sensitive, 10/14 strains (Table 1). Of the bacterial species for which there were more than ten strains in the panels, *E. cloacae* had 2/14, *E. coli* had 6/34, *K. pneumoniae* had 17/51, and *P. aeruginosa* had 6/12 strains that were not sensitive to the 1:1 combination of GT-1 and GT-055 (Table 1). The antibiotic resistance genes that are present in the strains in the CDC AR bank panels are known and shared on the CDC AR Isolate Bank website [15]. Ten genes associated with β-lactamase resistance were present in more than 10 strains of bacteria, and only one was associated with resistance to the GT-1 and GT-055 combination treatment in a majority of the strains. NDM-1 was present in 28 strains, and 23 of those were resistant to GT-1/GT-055 combination (Supplemental Table 1). About half of strains with OXA-1 (16/31), OXA-50 (6/12), PAO (6/12), and SHV-1 (9/18) were also resistant to the combination. Fewer than 50% of the strains with the other genes, CTX-M-15 (16/38), KPC3 (1/20), OXA-9 (9/27), TEM-1A (7/28), TEM-1B (12/41) were resistant to the combination.

### MICs of GT-1 and GT-055 against biothreat bacteria

The MICs of GT-1 alone or in combination with GT-055 against biothreat bacteria were determined in both standard cation-adjusted Mueller Hinton broth (CAMHB) and iron-depleted CAMHB (ID-CAMHB). When tested against representative strains, GT-1, GT-1:GT-055 (1:1), and GT-1 with GT-055 (fixed 4 μg ml^−1^) exhibited potent activity against *B. pseudomallei*, *Y. pestis*, and *F. tularensis*, were less active against *B. mallei*, and inactive against *B. anthracis* (Table 2). The MICs were lower for the bacteria grown in ID-CAMHB than those grown in CAMHB for *B. mallei* and *Y. pestis* and were similar between the conditions for *B. pseudomallei* and *F. tularensis*. The MIC_90_s were calculated after the MIC was determined for 30 strains of *B. mallei*, *B. pseudomallei*, and *Y. pestis* (Table 3). The MIC_90_ values against these strains mirrored what was seen with the representative strains; the MIC_90_s for *B. mallei* and *Y. pestis* were lower in the ID-CAMHB than they were in the CAMHB. In *B. mallei*, the MIC_90_s (ID-CAMHB vs CAMHB) were 8 vs 32 μg ml^−1^ for GT-1, 4/2 vs 8/4 μg ml^−1^ for GT-1:GT-055 (2:1), and 2/4 vs 8/4 μg ml^−1^ for GT-1 with a fixed 4 μg ml^−1^ concentration of GT-055. The *Y. pestis* MIC_90_s (ID-CAMHB vs CAMHB) were 0.5 vs 2 μg ml^−1^ for GT-1, 0.5/0.25 vs 2/1 μg ml^−1^ GT-1:GT-055 (2:1), and 0.12/4 vs 1/4 μg ml^−1^ for GT-1 with a fixed 4 μg ml^−1^ concentration of GT-055. The *B. pseudomallei* MIC_90_s were actually higher in the ID-CAMHB versus the CAMHB for GT-1 (0.12 vs ≤0.03 μg ml^−1^), GT-1 at 2:1 ratio with GT-055 (0.25/0.125 vs ≤0.03/≤0.015 μg ml^−1^), or GT-1 with a fixed concentration of GT-055 (0.06/4 vs ≤0.03/4 μg ml^−1^), but had low MIC_90_s for both conditions.

**Table 2:** MIC^a^ against representative strains of biothreat bacterial pathogens

	Media	*B. anthracis* Ames	*B. pseudomallei* K96243	*B. mallei* FMH	*F. tularensis* Schu4	*Y. pestis* CO92
GT-1	CAMHB	>128	≤0.03	16	2	2
	ID-CAMHB	>128	0.06	8	2	0.25
GT-1/GT-055 (2:1)	CAMHB	>128/64	≤0.013/0.015	16/8	2	1/0.5
	ID-CAMHB	>128/64	0.125/0.06	4/2	4	0.25/0.125
GT-1 + GT-055 (4 μg/ml)	CAMHB	>128/4	≤0.03/4	8/4	4/4	0.5/4
	ID-CAMHB	>128/4	0.125/4	1/4	4/4	0.12/4
GT-055	CAMHB	>64	>32	>64	>32	>32
	ID-CAMHB	>64	>64	64	32	>32
DOX	CAMHB	0.06	1	0.25	0.25	1
	ID-CAMHB	0.5	4	0.06	≤0.015	0.5

^a^MIC (μg/ml) of GT-1 or GT-055 or GT-1/GT-055 in combination

**Table 3:** MIC_90_ and range of MICs^a^ against biothreat bacterial pathogens

Compounds	Media	*B. pseudomallei*	*B. mallei*	*Y. pestis*
GT-1	CAMHB	≤0.03 (≤0.03-0.5)	32 (4–32)	2 (0.12–4)
	ID-CAMHB	0.12 (≤0.03–0.12)	8 (2–16)	0.5 (≤0.03–0.5)
GT-1/GT-055 (2:1)	CAMHB	≤0.03/≤0.015 (≤0.03/≤0.015–0.25/0.125)	8/4 (2/1–16/8)	2/1 (0.12/0.06–2/1)
	ID-CAMHB	0.25/0.125 (≤0.03/≤0.015–8/4)	4/2 (2/1–8/4)	0.5/0.25 (0.06/0.03–1/0.5)
GT-1 + GT-055 (4 μg/ml)	CAMHB	≤0.03/4 (≤0.03/4)	8/4 (1/4–16/4)	1/4 (≤0.03/4–2/4)
	ID-CAMHB	0.06/4 (≤0.03/4–4/4)	2/4 (0.5/4–4/4)	0.12/4 (≤0.03/4–0.25/4)
GT-055	CAMHB	>32 (>32)	>32 (32–>32)	>32 (8–>32)
	ID-CAMHB	>32 (>32)	>32 (>–32)	>32 (2–>32)
DOX	CAMHB	2 (0.25–2)	0.12 (≤0.015–0.25)	1 (0.12–1)
	ID-CAMHB	1 (0.5–>32)	0.25 (≤0.015–0.5)	1 (0.12–2)

^a^MIC (μg/ml) of GT-1 or GT-055 or GT-1/GT-055 in combination

### In vivo efficacy

BALB/c mice were challenged via aerosol with 4 × LD_50_ of *B. pseudomallei* strain 1026b (MIC ≤ 0.03 μg ml^−1^). A 21 day treatment regimen of GT-1 alone or in combination with GT-055 was initiated. Only one of the groups that received GT-1 alone or GT-1 and GT-055 (1:1 ratio) at 12 h post-infection had 10% survival, while the group that received 120/120 mg kg^−1^ GT-1/GT-055 exhibited 30% survival (Table 4). Mice had a median survival of 7.5–8.5 days for the three doses of GT-1 tested, 30, 60, and 120 mg kg^−1^. When mice received GT-055 with GT-1, the median survival was higher than that observed for mice that only received GT-1 with the largest increase occurring in the highest dose group: 7.5 days (GT-1, 30 mg kg^−1^) vs 10.5 days (GT-1/GT-055, 30/30 mg kg^−1^); 8.5 days (GT-1, 60 mg kg^−1^) vs 14.5 days (GT-1/GT-055, 60/60 mg kg^−1^); and 8 days (GT-1, 120 mg kg^−1^) vs 42.5 days (GT-1/GT-055, 120/120 mg kg^−1^). A similar increase in survival occurred when the highest dose was initiated at 6 h post-infection; the median survival of mice that received 120 mg kg^−1^ GT-1 or 120/120 mg kg^−1^ GT-1/GT-055 were 12 and 42.5 days, respectively. The bacterial burden was then determined in the spleens of mice that survived in each group. Bacteria were not detected in the spleens of the mice that received the 120/120 mg kg^−1^ GT-1/GT-055 dose initiated at 12 h post-infection, whereas the other survivors had enlarged spleens and at least 10^8^ CFU/spleen (Table 5). The bacteria present in the spleens of representative surviving animals treated with GT-1 or GT-1/GT-055 were tested for susceptibility to GT-1 and the combination, and no differences in MIC compared to the starting isolates were observed (data not shown), which makes it unlikely that the failure of GT-1 or the combination to clear the infections resulted from development of resistance to these agents.

**Table 4:** Summary of GT-1 and GT-1/GT-055 efficacy in mouse model of *B. pseudomallei* infection

Cohort	Antibiotic	Dose	# Deaths	Median survival (Days post-challenge)	% Survival
1	Saline	q6	10	6	0
2	Ceftazidime	300 mg/kg, q6	2	Undefined	80
3	GT-1	30 mg/kg, q6	10	7.5	0
4	GT-1	60 mg/kg, q6	10	8.5	0
5	GT-1	120 mg/kg, q6	10	8	0
6	GT-1	120 mg/kg, q6, 6-hour start	9	12	10
7	GT-1/GT-055	30/30 mg/kg, q6	9	10.5	10
8	GT-1/GT-055	60/60 mg/kg, q6	10	14.5	0
9	GT-1/GT-055	120/120 mg/kg, q6	7	42.5	30
10	GT-1/GT-055	120 mg/kg, q6, 6-hour start	9	42.5	10

**Table 5:** Bacterial load in spleens of *B. pseudomallei*-infected and treated mice that survived until day 60 post-infection

Treatment groups	Spleen weight (g)	*B. pseudomallei* count (CFU/spleen)
Ceftazidime 300 mg/kg, q6	0.089	0
	0.090	0
	0.084	0
	0.088	0
	0.088	0
	0.090	0
	0.092	0
	0.098	0
GT-1 120 mg/kg, q6, 6-hour start	0.719	1.80E + 08
GT-1/GT-055 30/30 mg/kg, q6	1.165	1.34E + 08
GT-1/GT-055 120/120 mg/kg, q6	0.099	0
	0.365	0
	0.355	0
GT-1/GT-055 120/120 mg/kg, q6, 6-hour start	1.889	9.50E + 08

**Fig. 2:**
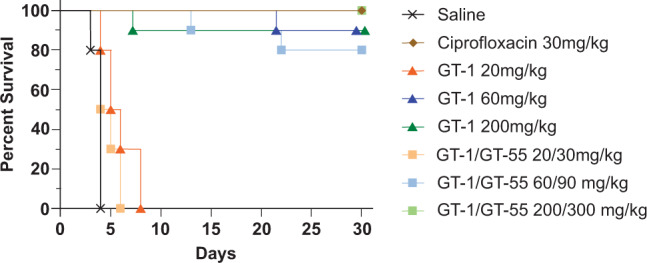
Efficacy of GT-1 and GT-1/GT-055 in *Y*. *pestis* challenge study. Mice infected with 110X LD_50_ of *Y. pestis* strain CO92. Treatment with saline (negative control), ciprofloxacin (30 mg kg^−1^, q12h), and GT-1 (q6h) or GT-1/GT-55 (q6h) at one of the three specified doses was initiated at 6 h post-infection and continued for 7 days (*n* = 10/group). Survival was monitored for 30 days

Efficacy of GT-1 with GT-055 was also assessed after an intranasal (IN) challenge of 110xLD_50_ of *Y. pestis*. Mice that received the lowest doses of either GT-1 (20 mg kg^−1^) or GT-1/GT-055 (20/30 mg kg^−1^) succumbed to the infection by day 7. When mice received GT-1 at either 60 or 200 mg kg^−1^, they had 90% survival while the combination of GT-1/GT-055 60/90 mg kg^−1^ or 200/300 mg kg^−1^ resulted in 80% or 100% survival, respectively (Fig. 2). The bacterial load in the lungs and spleens from 3 mice that survived in each group were determined. The spleens isolated from the surviving mice had no detectable bacteria, but at least one mouse from each group had bacteria present in the lungs (Table 6).

**Table 6:** Bacterial load in *Y. pestis* mice survivors

Treatment	Spleen weight (g)	CFU/g spleen	Lung weight (g)	CFU/g lung (×10^3^)
Ciprofloxacin 30 mg/kg	0.100	0	0.232	0
	0.107	0	0.313	16.0
	0.097	0	0.401	12.5
GT-1 60 mg/kg	0.103	0	0.383	13.1
	0.192	0	0.256	19.5
	0.088	0	0.356	0
GT-1 200 mg/kg	0.086	0	0.395	0.5
	0.107	0	0.526	0
	0.078	0	0.315	1.1
GT-1/GT-055 60/90 mg/kg	0.093	0	0.381	13.1
	0.087	0	0.237	21.1
	0.103	0	0.313	0
GT-1/GT-055 200/300 mg/kg	0.087	0	0.267	18.7
	0.073	0	0.332	0
	0.072	0	0.226	0

## Discussion

The incorporation of an iron-binding siderophore moiety with an antibiotic is designed to act as a “Trojan horse”, delivering an antibiotic to a bacterial pathogen via the siderophore-uptake machinery that many bacteria possess in order to obtain the iron needed for survival. This strategy, when incorporated with a novel antibiotic, provides another tool and potential solution to the problem of AMR in specific pathogens. Cefiderocol (formerly S-649266), a siderophore cephalosporin, uses this approach to penetrate through the outer membrane of Gram negative bacteria and thereby gain access to the penicillin binding protein targets of inhibition and is approved for use without a β-lactamase inhibitor [9].

GT-1 contains a siderophore fused to a novel cephalosporin to create such a molecule. Of the panel of nosocomial and community-acquired bacterial pathogens tested, the combination of GT-1 and a broad-spectrum BLI, GT-055, demonstrated the greatest in vitro efficacy versus *E. coli* and *E. cloacae*, with >80% of strains tested yielding sub-4 µg ml^−1^ MIC values. Of the 16 bacterial pathogens tested, 68% had sub-4 µg ml^−1^ MIC values. These results are consistent with the susceptibility of clinical isolates of the ESKAPE pathogens to GT-1 and GT-055 in previous studies [6, 8]. The presence of NDM-1 seemed to correlate with resistance in the strains tested here, as 23/28 strains with that enzyme were resistant to the combination. A previous study examined the β-lactamases present in the clinical isolates that were tested for susceptibility to GT-1 and/or GT-055, but NDM-1 was not represented [8]. When cefiderocol was tested against 49 NDM-1-positive *E. coli*, *K. pneumoniae*, and other Enterobacterales, only 5 *E. coli* strains were resistant to the antibiotic [7]. Further characterization of clinical isolates with NDM-1 would be required to determine if its presence would confer resistance to the GT-1/GT-055 combination.

The combination of GT-1 and GT-055 was particularly effective in vitro against the biothreat pathogens *B. pseudomallei* and *Y. pestis*. Cefiderocol has also demonstrated potent in vitro activity against these biothreat pathogens [22, 23], although there are no published studies of its in vivo activity against these pathogens in the mouse models of infection. Our present studies demonstrate that GT-1 alone or in combination with GT-055 has potent activity against *Y. pestis* in a murine model of intranasal infection. The highest dose of GT-1/GT-055 resulted in 100% survival of the mice, equivalent to the cohort treated with ciprofloxacin. When mice were examined for bacterial burden in the lungs at the end of study, one of three mice from the GT-1/GT-055 highest dose group had bacteria present, whereas two of three mice treated with ciprofloxacin had bacteria present. The highest doses of GT-1/GT-055 were comparable to ciprofloxacin in their ability to protect the mice post-infection and could be a feasible treatment options if those doses were achievable in patients.

We initiated treatment of the *Y. pestis*-infected mice at 6 h post-infection and saw at least 80% survival in the two highest doses of GT-1 alone and GT-1/GT-055. The use of β-lactam antibiotics to treat *Y. pestis* infections is contra-indicated. Previous mouse studies observed that mice infected via aerosol exposure to *Y. pestis* that received the β-lactam antibiotics cephalosporins, ampicillin, or aztreonam, at 42 h post-infection succumbed to infection faster than the mice that received a saline control [24]. These antibiotics were moderately effective when treatment was initiated at 24 h post-infection, with survival rates between 25 and 100% for different cephalosporins but were, for the most part, sub-optimal compared to the 100% survival observed after ciprofloxacin treatment. A similar decline in efficacy in later treatment initiation was observed in another study that investigated the efficacy of imipenem and ceftazidime as treatments after aerosol exposure to *Y. pestis* [25]. That study also analyzed the cytokine profiles and release of endotoxin after treatment initiation. The release of endotoxin after β-lactam treatment is a concern, but the ciprofloxacin treated-mice released more endotoxin than was seen after treatment with either ceftazidime or imipenem. The potential effect of ciprofloxacin on other toxins produced by *Y. pestis* was not examined. Treatment with any of the three antibiotics resulted in the release of less endotoxin than was observed in the saline-treated control group [25]. It is unclear if a later initiation time would have also resulted in a similar decline in efficacy of GT-1 or GT-1/GT-055, but it seems possible.

The combined treatment of GT-1/GT-055 increased the mean time until death in mice infected with *B. pseudomallei*. However, a bacterial load was still evident in the spleen of the few survivors in all but one group, GT-1/GT-055 120/120 mg kg^−1^. It is not clear why the very potent activity of GT-1 and GT-1/GT-055 against *B. pseudomallei* observed in vitro is not recapitulated in vivo in the mouse model. The doses tested may have failed to achieve the required concentrations of the drugs at the intracellular location of the bacteria, or differences in expression of iron-uptake systems may occur in vitro and in vivo, thus affecting uptake of GT-1 into the periplasmic space of the pathogen [26]. Additional studies would be required to better understand the basis for these findings. Given that less than 50% of mice survived and the continued bacterial presence in the spleen, suggests that GT-1/GT-055 is unlikely to be a viable treatment option for *B. pseudomallei* infections.

Here, we have demonstrated a broad-spectrum in vitro activity of GT-1 and GT-1/GT-055 combination treatment against MDR bacteria and biodefense pathogens. The in vitro activity did not translate into efficacy against an in vivo infection model of *B. pseudomallei*. However, both GT-1 alone and the GT-1/GT-055 combination were effective when administered soon after *Y. pestis* infection in mice.

## Supplementary information

Supplemental Table 1
