# Supplementary material for: In vitro and in vivo activity of GT-1, a novel siderophore cephalosporin, and GT-055, a broad-spectrum β-lactamase inhibitor, against biothreat and ESKAPE pathogens
Source: J Antibiot (Tokyo). 2021 Sep 14;74(12):884–92. doi: 10.1038/s41429-021-00472-9 (PMC8627911; doi:10.1038/s41429-021-00472-9)
Supplement: Supplementary file 1 — Supplemental Table 1 [file 41429_2021_472_MOESM1_ESM.docx]

Supplemental Table 1. Summary of CDC Panel MIC and β-lactamase genes present

| **CDC AR Bank** | **Strain** | **GT-1** | **GT-1 + GT-055 1:1^a^** | **GT-1 + GT-055 2:1^a^** | **GT-055** | **CAZ/ AVI^b^** | **Beta-lactamases^c^** |
| --- | --- | --- | --- | --- | --- | --- | --- |
| **Enterobacteriaceae Carbapenem Breakpoint** | | | | | | | |
| 0001 | *Escherichia coli* | 0.5 | 0.25 | 0.5 | > 32 | > 32 | KPC-3, **OXA-1** |
| 0002 | *Enterobacter cloacae* | 32 | 4 | 4 | > 32 | > 32 | KPC-3, OXA-9, TEM-1A |
| 0003 | *Klebsiella pneumoniae* | 0.5 | 0.5 | 0.5 | > 32 | 1 | KPC-3, OXA-9, SHV-12 |
| 0004 | *Klebsiella pneumoniae* | 4 | 2 | 4 | > 32 | 8 | KPC-3, OXA-9, TEM-1A, **SHV-11** |
| 0005 | *Klebsiella pneumoniae* | 32 | 1 | 4 | > 32 | 32 | KPC-2, OXA-9, TEM-1A, **SHV-11** |
| 0006 | *Escherichia coli* | > 32 | 2 | 4 | > 32 | > 32 | TEM-1B, CMY-2, CTX-M-14 |
| 0007 | *Enterobacter aerogenes* | > 32 | 16 | 16 | > 32 | 32 |  |
| 0008 | *Enterobacter cloacae* | 8 | 2 | 2 | > 32 | 16 | ACT-15 |
| 0009 | *Enterobacter aerogenes* | > 32 | 2 | 4 | > 32 | > 32 |  |
| 0010 | *Klebsiella pneumoniae* | 4 | 1 | 0.5 | 16 | 2 | CMY-94, SHV-1 |
| 0011 | *Escherichia coli* | > 32 | 1 | 2 | 8 | 1 | CTX-M-15, **OXA-1** |
| 0012 | *Klebsiella pneumoniae* | > 32 | 2 | 2 | > 32 | 8 | SHV-12 |
| 0013 | *Escherichia coli* | 4 | 0.5 | 0.5 | 1 | ≤ 0.063 | TEM-1B, CTX-M-14 |
| 0014 | *Escherichia coli* | > 32 | 1 | 1 | 2 | > 32 | CTX-M-15, **OXA-1** |
| 0015 | *Escherichia coli* | > 32 | 1 | 2 | 16 | 1 | TEM-1B, CTX-M-15, **OXA-1** |
| 0016 | *Klebsiella pneumoniae* | 0.125 | 0.25 | 0.125 | > 32 | 0.25 | LEN16 |
| 0017 | *Escherichia coli* | > 32 | > 32 | > 32 | > 32 | 4 |  |
| 0018 | *Enterobacter aerogenes* | 0.25 | 0.5 | 0.25 | > 32 | 0.125 |  |
| 0019 | *Escherichia coli* | 16 | 0.5 | 1 | 0.5 | ≤ 0.063 | TEM-1B |
| 0020 | *Escherichia coli* | 4 | 8 | 8 | > 32 | 0.25 |  |
| 0021 | *Citrobacter freundii* | 2 | 0.125 | 0.5 | 2 | 0.5 | CMY-80 |
| 0022 | *Citrobacter freundii* | ≤ 0.063 | ≤ 0.063 | ≤ 0.063 | 2 | ≤ 0.063 | CMY-84 |
| 0023 | *Citrobacter freundii* | 2 | 32 | 2 | > 32 | > 32 | CMY-80 |
| 0024 | *Citrobacter koseri* | 32 | 0.5 | 1 | 2 | 0.25 | MAL-1 |
| 0025 | *Citrobacter koseri* | 2 | 1 | 0.5 | 4 | 0.5 | TEM-1B, MAL-1 |
| 0026 | *Providencia stuartii* | 0.5 | 1 | 0.5 | > 32 | 0.125 |  |
| 0027 | *Serratia marcescens* | 0.25 | > 32 | > 32 | > 32 | > 32 |  |
| 0028 | *Klebsiella oxytoca* | ≤ 0.063 | 0.125 | ≤ 0.063 | > 32 | 0.125 |  |
| 0029 | *Proteus mirabilis* | 0.25 | 1 | 0.5 | > 32 | ≤ 0.063 |  |
| 0030 | *Shigella sonnei* | 2 | 0.25 | 2 | 2 | 0.25 | TEM-1B |
| 0031 | *Salmonella typhimurium* | ≤ 0.063 | ≤ 0.063 | ≤ 0.063 | 8 | 0.25 |  |
| **Gram-negative Carbapenemase Detection Panel** | | | | | | | |
| 0032 | *Enterobacter cloacae* | 0.25 | 0.5 | 0.25 | 32 | 32 | KPC-3, TEM-1B, ACT-16 |
| 0033 | *Acinetobacter baumannii* | > 32 | > 32 | > 32 | > 32 | > 32 | **NDM-1**, OXA-94 |
| 0034 | *Klebsiella pneumoniae* | > 32 | 2 | 4 | > 32 | > 32 | IMP-4, TEM-1B, **SHV-11** |
| 0035 | *Acinetobacter baumannii* | 32 | > 32 | > 32 | > 32 | > 32 | TEM-1D, ADC-25, OXA-66, OXA-72 |
| 0036 | *Acinetobacter baumannii* | 2 | 2 | 2 | > 32 | > 32 | OXA-65, OXA-24 |
| 0037 | *Acinetobacter baumannii* | > 32 | > 32 | > 32 | > 32 | > 32 | **NDM-1**, OXA-94 |
| 0038 | *Enterobacter cloacae* | > 32 | 8 | 16 | > 32 | > 32 | **NDM-1**, OXA-9, TEM-1B, ACT-7, CTX-M-15, **OXA-1** |
| 0039 | *Klebsiella pneumoniae* | > 32 | 1 | 4 | > 32 | 2 | **OXA-1**81, CTX-M-15, SHV-26 |
| 0040 | *Klebsiella pneumoniae* | > 32 | 4 | 32 | > 32 | > 32 | VIM-27, CTX-M-15, **SHV-11**, **OXA-1** |
| 0041 | *Klebsiella pneumoniae* | > 32 | > 32 | > 32 | > 32 | > 32 | **NDM-1**, CMY-4, CTX-M-15, **SHV-11**, **OXA-1**0 |
| 0042 | *Klebsiella pneumoniae* | > 32 | 1 | 2 | > 32 | > 32 | TEM-1B, CTX-M-15, SHV-1, **OXA-1**0, **OXA-1** |
| 0043 | *Klebsiella pneumoniae* | 0.25 | 0.25 | 0.125 | 4 | 1 | SHV-12 |
| 0044 | *Klebsiella pneumoniae* | > 32 | 2 | 4 | > 32 | 8 | OXA-9, TEM-1A, CTX-M-15, SHV-12, **OXA-1** |
| 0045 | *Acinetobacter baumannii* | 8 | 2 | 16 | > 32 | > 32 | TEM-1D, OXA-23, OXA-69 |
| 0046 | *Klebsiella pneumoniae* | > 32 | > 32 | > 32 | > 32 | > 32 | VIM-27, CTX-M-15, **SHV-11**, **OXA-1** |
| 0047 | *Klebsiella pneumoniae* | > 32 | 4 | 8 | > 32 | 16 | TEM-1A |
| 0048 | *Escherichia coli* | > 32 | 4 | 8 | 4 | > 32 | **NDM-1**, TEM-1B, CMY-6, CTX-M-15, OXA-2 |
| 0049 | *Klebsiella pneumoniae* | > 32 | > 32 | > 32 | > 32 | > 32 | **NDM-1**, TEM-1B, CMY-6, CTX-M-15, **OXA-1** |
| 0050 | *Enterobacter cloacae* | 32 | 2 | 4 | > 32 | 4 | KPC-4, TEM-1A, ACT-5 |
| 0051 | *Klebsiella ozaenae* | > 32 | 1 | 1 | > 32 | 1 | **OXA-1**81, CTX-M-15, SHV-26 |
| 0052 | *Acinetobacter baumannii* | 1 | 1 | 1 | > 32 | 16 | OXA-58, **OXA-1**00 |
| 0053 | *Enterobacter cloacae* | > 32 | 2 | 4 | 32 | 4 | KPC-3, OXA-9, TEM-1A |
| 0054 | *Pseudomonas aeruginosa* | 8 | 8 | 16 | > 32 | > 32 | VIM-4, **OXA-50**, **PAO** |
| 0055 | *Escherichia coli* | > 32 | > 32 | > 32 | > 32 | > 32 | **NDM-1**, CMY-6, **OXA-1** |
| 0056 | *Acinetobacter baumannii* | > 32 | 32 | > 32 | > 32 | > 32 | OXA-23, OXA-66 |
| 0057 | *Morganella morganii* | > 32 | > 32 | > 32 | > 32 | > 32 | **NDM-1**, CTX-M-15, **OXA-1** |
| 0058 | *Escherichia coli* | 8 | 1 | 2 | > 32 | 0.25 | TEM-52B |
| 0059 | *Proteus mirabilis* | 32 | 32 | 32 | > 32 | 2 | TEM-1B |
| 0060 | *Enterobacter cloacae* | > 32 | 2 | 4 | 4 | > 32 | ACT-7 |
| 0061 | *Escherichia coli* | 0.25 | ≤ 0.063 | ≤ 0.063 | 8 | > 32 | KPC-3, OXA-9, TEM-1A |
| 0062 | *Enterobacter aerogenes* | 32 | 1 | 2 | > 32 | 2 |  |
| 0063 | *Acinetobacter baumannii* | > 32 | > 32 | > 32 | > 32 | > 32 | OXA-23, OXA-24, OXA-65 |
| 0064 | *Pseudomonas aeruginosa* | > 32 | > 32 | > 32 | > 32 | > 32 | SPM-1, **OXA-50**, **PAO**, OXA-56 |
| 0065 | *Enterobacter cloacae* | 32 | 1 | 2 | 4 | 0.5 | ACT-15 |
| 0066 | *Klebsiella pneumoniae* | > 32 | 2 | 8 | > 32 | 2 | OXA-232, OXA-9, TEM-1A, CTX-M-15, **OXA-1** |
| 0067 | *Escherichia coli* | 0.25 | 1 | 0.5 | 2 | 0.25 | TEM-1B |
| 0068 | *Klebsiella pneumoniae* | > 32 | > 32 | > 32 | > 32 | > 32 | **NDM-1**, OXA-232, OXA-9, TEM-1A, CTX-M-15, **SHV-11**, **OXA-1** |
| 0069 | *Escherichia coli* | > 32 | 1 | 4 | 2 | > 32 | **NDM-1**, TEM-1B, CMY-6 |
| 0070 | *Acinetobacter baumannii* | 2 | 1 | 1 | > 32 | 16 | OXA-58, **OXA-1**00 |
| 0071 | *Klebsiella oxytoca* | 16 | 4 | 2 | > 32 | 2 | OXY-2-8 |
| 0072 | *Enterobacter cloacae* | > 32 | 2 | 2 | 4 | > 32 | TEM-1B |
| 0073 | *Enterobacter cloacae* | > 32 | 1 | 2 | 2 | 4 |  |
| 0074 | *Enterobacter aerogenes* | 1 | 0.125 | ≤ 0.063 | 32 | 0.25 | OXA-48 |
| 0075 | *Klebsiella pneumoniae* | > 32 | 2 | 4 | 32 | 2 | OXA-232, CTX-M-15, SHV-1, **OXA-1** |
| 0076 | *Klebsiella pneumoniae* | > 32 | 16 | 4 | > 32 | > 32 | VIM-1, SHV-30 |
| 0077 | *Escherichia coli* | 0.125 | 0.125 | ≤ 0.063 | 2 | 0.125 |  |
| 0078 | *Acinetobacter baumannii* | 32 | 16 | 32 | > 32 | > 32 | ADC-25, SHV-5, OXA-71 |
| 0079 | *Klebsiella pneumoniae* | 4 | 1 | 1 | 32 | 2 | TEM-1B, CTX-M-14, **SHV-11**, DHA-1 |
| 0080 | *Klebsiella pneumoniae* | > 32 | 16 | 32 | > 32 | > 32 | IMP-4, TEM-1B, OKP-B-2, **OXA-1**, SFO-1 |
| 0081 | *Escherichia coli* | 32 | 1 | 2 | 4 | 0.25 | TEM-1B, CMY-2 |
| 0082 | *Providencia rettgeri* | > 32 | 16 | 32 | > 32 | > 32 | **NDM-1** |
| 0083 | *Acinetobacter baumannii* | > 32 | > 32 | > 32 | > 32 | > 32 | **NDM-1**, PER-7, OXA-23, OXA-69 |
| 0084 | *Escherichia coli* | 0.5 | 0.5 | 0.5 | > 32 | 1 | TEM-1B |
| 0085 | *Escherichia coli* | > 32 | 2 | 4 | 4 | > 32 | CMY-2 |
| 0086 | *Escherichia coli* | 1 | 1 | 1 | 2 | 0.125 | TEM-1B, CTX-M-14 |
| 0087 | *Klebsiella pneumoniae* | 32 | 1 | 4 | > 32 | 4 | SHV-12 |
| 0088 | *Acinetobacter baumannii* | > 32 | > 32 | > 32 | > 32 | > 32 | **NDM-1**, OXA-64 |
| 0089 | *Escherichia coli* | 32 | 1 | 1 | > 32 | 0.5 | CMY-2 |
| 0090 | *Pseudomonas aeruginosa* | 0.5 | 0.5 | 0.5 | > 32 | 8 | KPC-5, **OXA-50**, **PAO** |
| 0091 | *Serratia marcescens* | 0.125 | 0.25 | 0.25 | > 32 | 0.5 | SME-3 |
| 0092 | *Pseudomonas aeruginosa* | > 32 | 32 | 32 | > 32 | > 32 | IMP-14, **OXA-50**, VEB-1, **PAO**, **OXA-1**0 |
| 0093 | *Enterobacter cloacae* | 32 | 1 | 8 | 16 | 8 | KPC-6, TEM-1B, ACT-16, **OXA-1** |
| 0094 | *Pseudomonas aeruginosa* | 1 | 0.25 | 0.25 | > 32 | > 32 | **OXA-50**, **PAO** |
| 0095 | *Pseudomonas aeruginosa* | 4 | 4 | 8 | > 32 | 8 | **OXA-50**, **PAO** |
| 0096 | *Klebsiella ozaenae* | 32 | 2 | 4 | > 32 | > 32 | KPC-3, OXA-9, TEM-1A, SHV-1 |
| 0097 | *Klebsiella pneumoniae* | 16 | 2 | 4 | > 32 | 2 | KPC-3, OXA-9, TEM-1A, **SHV-11** |
| 0098 | *Klebsiella pneumoniae* | > 32 | 4 | 8 | 32 | > 32 | KPC-2, OXA-9, TEM-1A |
| 0099 | *Serratia marcescens* | 0.5 | 0.25 | 0.125 | > 32 | 0.125 | SME-3 |
| 0100 | *Pseudomonas aeruginosa* | 16 | 16 | 16 | > 32 | > 32 | VIM-2, **OXA-50**, **PAO** |
| 0101 | *Acinetobacter baumannii* | 16 | 32 | 16 | > 32 | > 32 | OXA-65, OXA-24 |
| 0102 | *Acinetobacter baumannii* | 32 | 32 | 32 | > 32 | > 32 | ADC-25, OXA-66 |
| 0103 | *Pseudomonas aeruginosa* | 32 | > 32 | 32 | > 32 | > 32 | IMP-1, **OXA-50**, **PAO** |
| 0104 | *Escherichia coli* | 32 | 1 | 2 | 4 | 8 | KPC-4, TEM-1A |
| 0105 | *Pseudomonas aeruginosa* | 0.125 | 0.125 | 0.25 | > 32 | 32 | **OXA-50**, **PAO**, OXA-2 |
| 0106 | *Klebsiella pneumoniae* | > 32 | > 32 | > 32 | > 32 | > 32 | **NDM-1**, OXA-9, TEM-1A, CTX-M-15, **OXA-1** |
| 0107 | *Klebsiella pneumoniae* | 2 | 2 | 2 | > 32 | 2 | OXA-9, TEM-1A, SHV-83, CTX-M-2, **OXA-1**0 |
| 0108 | *Pseudomonas aeruginosa* | > 32 | > 32 | > 32 | > 32 | > 32 | VIM-2, **OXA-50**, **PAO**, OXA-4 |
| 0109 | *Klebsiella pneumoniae* | > 32 | 4 | 8 | > 32 | 0.25 | TEM-1B, CTX-M-15, **SHV-11**, **OXA-1** |
| 0110 | *Pseudomonas aeruginosa* | 1 | 1 | 2 | > 32 | > 32 | VIM-2, **OXA-50**, **PAO** |
| 0111 | *Pseudomonas aeruginosa* | 2 | 2 | 16 | > 32 | 32 | VIM-2, **OXA-50**, **PAO**, OXA-4 |
| **Enterobacteriaceae Carbapenemase Diversity Panel** | | | | | | | |
| 0112 | *Klebsiella pneumoniae* | 32 | 1 | 2 | > 32 | 2 |  |
| 0113 | *Klebsiella pneumoniae* | > 32 | 4 | 8 | > 32 | > 32 | KPC-3, **SHV-11** |
| 0114 | *Escherichia coli* | 32 | 4 | 8 | > 32 | > 32 | KPC-3, TEM-1B |
| 0115 | *Klebsiella pneumoniae* | 16 | 0.5 | 2 | 2 | 16 | KPC-3, TEM-1A |
| 0116 | *Citrobacter freundii* | > 32 | 4 | 8 | > 32 | 1 | KPC-2, CMY-79, CMY-76 |
| 0117 | *Klebsiella pneumoniae* | 32 | 2 | 4 | > 32 | 8 | KPC-3, OXA-9, TEM-1A |
| 0118 | *Escherichia coli* | > 32 | 2 | 4 | 4 | > 32 | **NDM-1**, TEM-1A, CMY-6, OXA-2 |
| 0119 | *Escherichia coli* | > 32 | 2 | 8 | 4 | > 32 | **NDM-1**, OXA-9, TEM-1B, CMY-6, CTX-M-15, **OXA-1**, OXA-2 |
| 0120 | *Klebsiella pneumoniae* | 32 | 2 | 4 | > 32 | 8 | KPC-2, TEM-1D |
| 0121 | *Serratia marcescens* | 2 | 0.5 | 0.5 | > 32 | 0.125 | SME-3 |
| 0122 | *Serratia marcescens* | 1 | 0.25 | 2 | > 32 | 0.125 | SME-3 |
| 0123 | *Serratia marcescens* | 0.25 | 0.5 | 1 | > 32 | 0.125 | SME-3 |
| 0124 | *Serratia marcescens* | 2 | > 32 | > 32 | > 32 | > 32 | SME-3 |
| 0125 | *Klebsiella pneumoniae* | 32 | 1 | 4 | > 32 | 1 | KPC-3, OXA-9, TEM-1B |
| 0126 | *Klebsiella pneumoniae* | ≤ 0.063 | ≤ 0.063 | 0.125 | > 32 | 0.5 | KPC-2, TEM-1B, **OXA-1** |
| 0127 | *Salmonella senftenberg* | > 32 | 32 | 32 | > 32 | > 32 | **NDM-1**, TEM-1B, CMY-4 |
| 0128 | *Escherichia coli* | > 32 | 4 | 8 | 4 | > 32 | **NDM-1**, CMY-6, CTX-M-15, OXA-2 |
| 0129 | *Klebsiella pneumoniae* | 32 | 1 | 2 | > 32 | 2 | KPC-3, TEM-1A |
| 0130 | *Serratia marcescens* | 1 | 0.5 | 0.5 | > 32 | 0.25 | SME-3 |
| 0131 | *Serratia marcescens* | 0.5 | 0.25 | 0.25 | > 32 | 0.125 | SME-3 |
| 0132 | *Enterobacter cloacae group* | 4 | 1 | 2 | 8 | 0.5 | NMC-A |
| 0133 | *Morganella morganii* | 32 | 8 | 8 | > 32 | 1 | KPC-2 |
| 0134 | *Raoultella ornithinolytica* | ≤ 0.063 | ≤ 0.063 | ≤ 0.063 | 8 | 0.5 | KPC-3, OXA-9, TEM-1A |
| 0135 | *Klebsiella pneumoniae* | > 32 | 2 | 4 | > 32 | > 32 | VIM-1, OXA-9, TEM-1A, SHV-12 |
| 0136 | *Enterobacter cloacae* | 0.25 | ≤ 0.063 | 0.125 | > 32 | 16 | KPC-3, OXA-9, TEM-1A, SHV-12 |
| 0137 | *Escherichia coli* | > 32 | 32 | 32 | 32 | > 32 | NDM-6, OXA-9, TEM-1A, CMY-42, CTX-M-15, **OXA-1** |
| 0138 | *Klebsiella pneumoniae* | > 32 | > 32 | > 32 | > 32 | > 32 | NDM-7, TEM-1B, CTX-M-15, **SHV-11** |
| 0139 | *Klebsiella pneumoniae* | > 32 | > 32 | > 32 | > 32 | > 32 | **NDM-1**, CMY-4, CTX-M-15, **SHV-11**, **OXA-1**0 |
| 0140 | *Klebsiella pneumoniae* | > 32 | 0.5 | 2 | > 32 | 0.5 | **OXA-1**81, CTX-M-15, SHV-26 |
| 0141 | *Klebsiella pneumoniae* | > 32 | 1 | 2 | > 32 | 2 | **OXA-1**81, CTX-M-15, SHV-26 |
| 0142 | *Klebsiella pneumoniae* | > 32 | 1 | 2 | > 32 | 0.5 | **OXA-1**81, CTX-M-15, SHV-26 |
| 0143 | *Klebsiella pneumoniae* | > 32 | > 32 | > 32 | > 32 | > 32 | **NDM-1**, OXA-9, TEM-1A, CMY-4, CTX-M-15 |
| 0144 | *Kluyvera ascorbata* | ≤ 0.063 | ≤ 0.063 | 0.25 | 2 | 1 | KPC-3, TEM-1B, CTX-M-124 |
| 0145 | *Klebsiella pneumoniae* | > 32 | > 32 | > 32 | > 32 | > 32 | **NDM-1**, OXA-9, TEM-1A, CTX-M-15, **SHV-11**, **OXA-1** |
| 0146 | *Klebsiella pneumoniae* | > 32 | > 32 | > 32 | > 32 | > 32 | **NDM-1**, CTX-M-15, **SHV-11**, **OXA-1** |
| 0147 | *Klebsiella oxytoca* | 2 | 1 | 2 | > 32 | 16 | KPC-3, OXY-1-4 |
| 0148 | *Klebsiella pneumoniae* | > 32 | > 32 | > 32 | > 32 | > 32 | **NDM-1**, TEM-1B, CMY-6, CTX-M-15, **SHV-11**, **OXA-1** |
| 0149 | *Escherichia coli* | > 32 | > 32 | > 32 | > 32 | > 32 | NDM-7, CMY-42 |
| 0150 | *Escherichia coli* | > 32 | 2 | 8 | > 32 | > 32 | NDM-5, TEM-1B, CMY-42 |
| 0151 | *Escherichia coli* | > 32 | 2 | 4 | 4 | > 32 | NDM-5, TEM-1B, CMY-42, CTX-M-15, SHV-12, **OXA-1** |
| 0152 | *Klebsiella pneumoniae* | > 32 | > 32 | > 32 | > 32 | > 32 | **NDM-1**, OXA-9, TEM-1A, CTX-M-15, **SHV-11**, **OXA-1** |
| 0153 | *Klebsiella pneumoniae* | > 32 | > 32 | > 32 | > 32 | > 32 | **NDM-1**, OXA-232, OXA-9, TEM-1A, CTX-M-15, **OXA-1** |
| 0154 | *Enterobacter cloacae* | > 32 | 32 | > 32 | > 32 | > 32 | VIM-1, TEM-1B, ACT-7 |
| 0155 | *Proteus mirabilis* | 8 | 1 | 4 | 16 | 0.125 | KPC-6 |
| 0156 | *Proteus mirabilis* | 1 | > 32 | > 32 | > 32 | ≤ 0.063 | KPC-2, **OXA-1**0 |
| 0157 | *Citrobacter species* | > 32 | > 32 | > 32 | > 32 | > 32 | **NDM-1**, OXA-9, TEM-1B, CTX-M-15 |
| 0158 | *Klebsiella pneumoniae* | > 32 | > 32 | > 32 | > 32 | > 32 | **NDM-1**, TEM-1B, CTX-M-15, **OXA-1** |
| 0159 | *Proteus mirabilis* | > 32 | > 32 | > 32 | > 32 | > 32 | **NDM-1** |
| 0160 | *Klebsiella pneumoniae* | 8 | 0.5 | 0.5 | > 32 | 0.25 | OXA-48, **SHV-11** |
| 0161 | *Enterobacter aerogenes* | > 32 | 16 | 8 | > 32 | > 32 | IMP-4, TEM-1B, **OXA-1**, SFO-1 |
| 0162 | *Escherichia coli* | > 32 | 4 | 16 | 8 | > 32 | NDM-7, TEM-1B, CTX-M-15 |
| 0163 | *Enterobacter cloacae* | > 32 | 1 | 2 | 2 | > 32 | KPC-2, TEM-1B, ACT-7, CTX-M-15, **OXA-1** |
| 0164 | *Enterobacter cloacae complex* | > 32 | 2 | 4 | > 32 | 32 | NMC-A |
| **Isolates with New or Novel Antibiotic Resistance** | | | | | | | |
| 0346 | *Escherichia coli* | > 32 | 2 | 4 | > 32 | 0.5 | CMY-2, CTX-M-55 |
| 0347 | *Klebsiella pneumoniae* | 16 | 8 | 8 | > 32 | > 32 | KPC-3 |
| 0348 | *Escherichia coli* | > 32 | 8 | 32 | 32 | > 32 | TEM-1B, CMY-2 |
| 0349 | *Escherichia coli* | > 32 | 0.25 | 1 | > 32 | > 32 | TEM-1B, CTX-M-14, CTX-M-55 |

^a^ MIC is concentration of GT-1

^b^ CAZ with AVI (4 μg/m); MIC is concentration of CAZ

^c^ Beta-lactamase genes in bold have MIC ≥ 8 μg/ml in at least 50% of strains with that gene
